# SNiPlay3: a web-based application for exploration and large scale analyses of genomic variations

**DOI:** 10.1093/nar/gkv351

**Published:** 2015-06-03

**Authors:** Alexis Dereeper, Felix Homa, Gwendoline Andres, Guilhem Sempere, Gautier Sarah, Yann Hueber, Jean-François Dufayard, Manuel Ruiz

**Affiliations:** 1 UMR Interaction Plante-Microorganismes et Environnement (IPME), Institut de Recherche pour le Développement (IRD), BP 64501, 34394 Montpellier Cedex 5, France; 2 UMR Amélioration Génétique et Adaptation des Plantes Méditerranéennes et Tropicales (AGAP), CIRAD, F-34398 Montpellier, France; 3 UPMC, CNRS, ABiMS Station Biologique, 29680 Roscoff, France; 4 UMR Intertryp CIRAD, Campus International de Baillarguet, 34398 Montpellier Cedex 5, France; 5 Bioversity International, Parc Scientifique Agropolis II, 34397 Montpellier Cedex 5, France; 6 Agrobiodiversity research area, International Center for Tropical Agriculture (CIAT), Cali 6713, Colombia

## Abstract

SNiPlay is a web-based tool for detection, management and analysis of genetic variants including both single nucleotide polymorphisms (SNPs) and InDels. Version 3 now extends functionalities in order to easily manage and exploit SNPs derived from next generation sequencing technologies, such as GBS (genotyping by sequencing), WGRS (whole gre-sequencing) and RNA-Seq technologies. Based on the standard VCF (variant call format) format, the application offers an intuitive interface for filtering and comparing polymorphisms using user-defined sets of individuals and then establishing a reliable genotyping data matrix for further analyses. Namely, in addition to the various scaled-up analyses allowed by the application (genomic annotation of SNP, diversity analysis, haplotype reconstruction and network, linkage disequilibrium), SNiPlay3 proposes new modules for GWAS (genome-wide association studies), population stratification, distance tree analysis and visualization of SNP density. Additionally, we developed a suite of Galaxy wrappers for each step of the SNiPlay3 process, so that the complete pipeline can also be deployed on a Galaxy instance using the Galaxy ToolShed procedure and then be computed as a Galaxy workflow. SNiPlay is accessible at http://sniplay.southgreen.fr.

## INTRODUCTION

Single nucleotide polymorphisms (SNPs) are genetic variants commonly used to identify candidate genes and genotype-phenotype association studies. With next generation sequencing (NGS), genome sequencing is becoming inexpensive and routine, and the discovery of large numbers of SNPs is facilitated. Indeed, with the availability of reference genome along with sequencing data derived from WGRS (whole-genome re-sequencing), GBS (genotyping by sequencing), RAD-Seq and RNA-Seq technologies, millions of variants including SNPs are easily released. To make exploration and large scale analyses of genomic variations simple and accessible, there is a need for applications based on efficient databases and convivial interfaces. Most of the existing tools are command-line (1) or dedicated to one type of analysis like GWAS (genome-wide association studies) (2,3) or phylogeny (4).

Here we report the version 3 of the SNiPlay application (5) that shows significant improvements for managing next generation data in terms of data filtering, analysis and visualization. Indeed, we improved the performance of SNiPlay for filtering large NGS datasets in a few seconds and for providing genome-wide analyses and visualizations. In addition to the previous analyses allowed by the application (genomic annotation of SNP, diversity analysis, haplotype reconstruction and network, linkage disequilibrium), SNiPlay3 proposes new modules for GWAS, population stratification, distance tree analysis and visualization of SNP density. To the best of our knowledge, no other web application allows the integration of a so large set of analyses from massive genotyping data at the whole-genome level.

## PROCESS OVERVIEW

The SNiPlay pipeline components have been updated to be able (i) to manage variants data derived from NGS technology and (ii) to process data at the whole-genome scale. One significant improvement is the ability to handle the standard VCF format (variant call format) as input files. Indeed, with the recent emergence of powerful software packages dedicated to analysis of NGS data such as VCFtools (6) or Snpeff (7) it has become possible to offer biologists an efficient complete analysis of a massive dataset at once in a few minutes.

**Figure 1.**
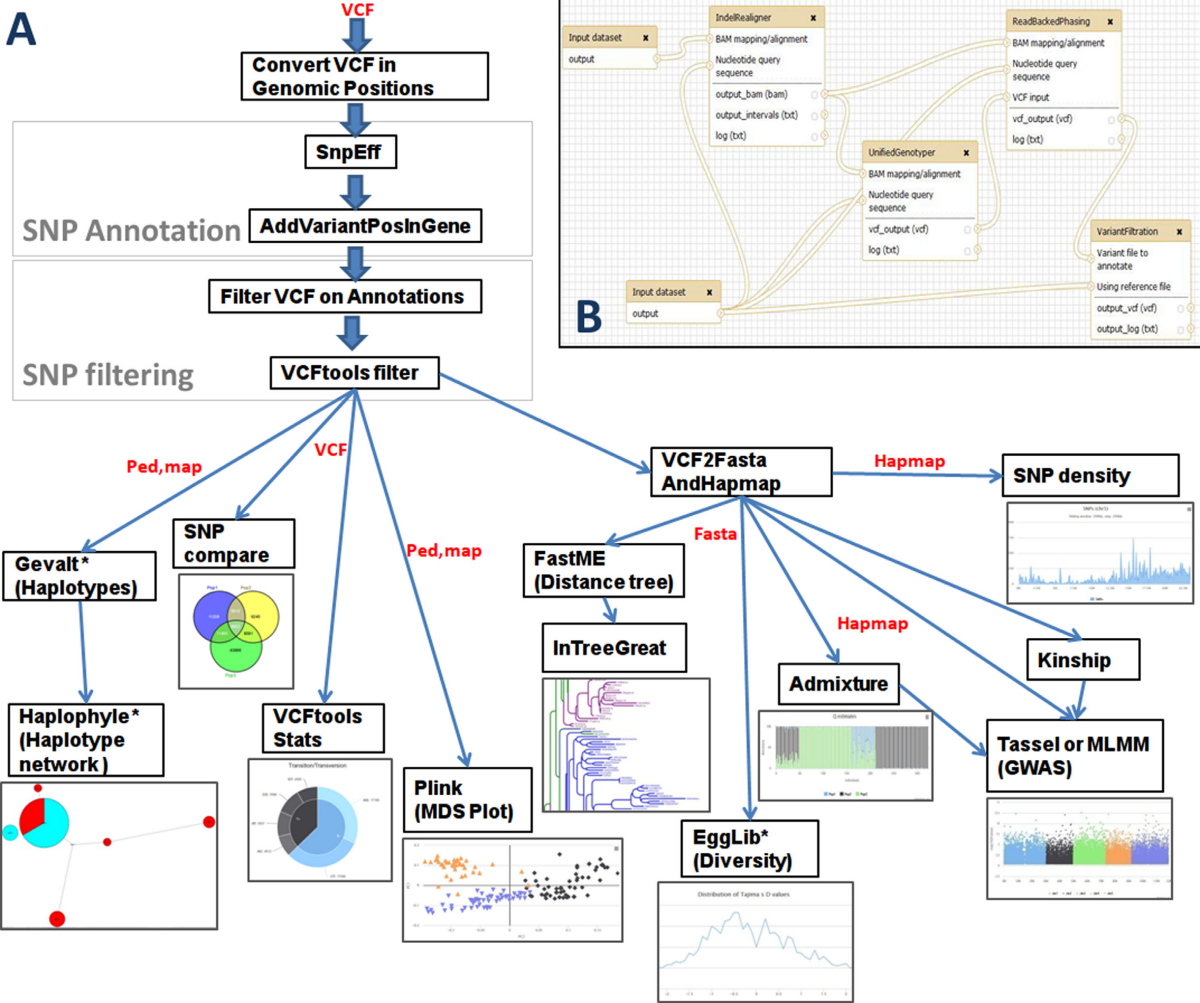
Overview of the SNiPlay3 process. (**A**) General schema of the process and graphical layouts of the different modules. For modules marked with an asterisk, analyses are computed gene by gene. Input and output file formats are indicated in red. (**B**) One of the SNiPlay Galaxy workflows: the SNP calling workflow based on the GATK package for data pre-processing.

An overview of the process is presented Figure 1A. The application offers numerous functionalities with attractive display layouts including GWAS, population structure, haplotype and linkage disequilibrium (LD) analyses, diversity analysis, SNP comparison between groups and general statistics about polymorphisms. Starting from a VCF file as entry point, the process first annotates the variants using an annotated reference genome to produce a new VCF file from which variants and genotyping data can be then mined and sent into a series of modules in charge of various processes. User has then the possibility to analyze variants either at the genome level or at the gene level. Most of the modules process genome-wide studies except for haplotype analyses—successively powered by the Gevalt software (8) for haplotype reconstruction and Haplophyle (9) for haplotype network—for which the analysis is done gene by gene or for user-defined genomic regions if do not exceed 200 variants (up to 200 regions). In this latter case, genes can be selected or directly provided as a list, while genomic regions can be defined by entering the limits, the application will loop and process these regions.

The different analyses are proposed to be computed either for a dataset directly uploaded by a user or for a genotyping dataset already available in the server as a VCF flat file associated to an annotated genome.

All tools and workflows are incorporated into the Galaxy framework, one of the most popular analysis platforms for NGS data, that offers easy access to numerous bioinformatic applications and strongly supports reproducibility of analysis steps (10–12). In addition to the pipelines offered through the website, our Galaxy workflows also include a pre-processing pipeline for SNP calling based on the GATK package (13) (Figure 1B).

**Figure 2.**
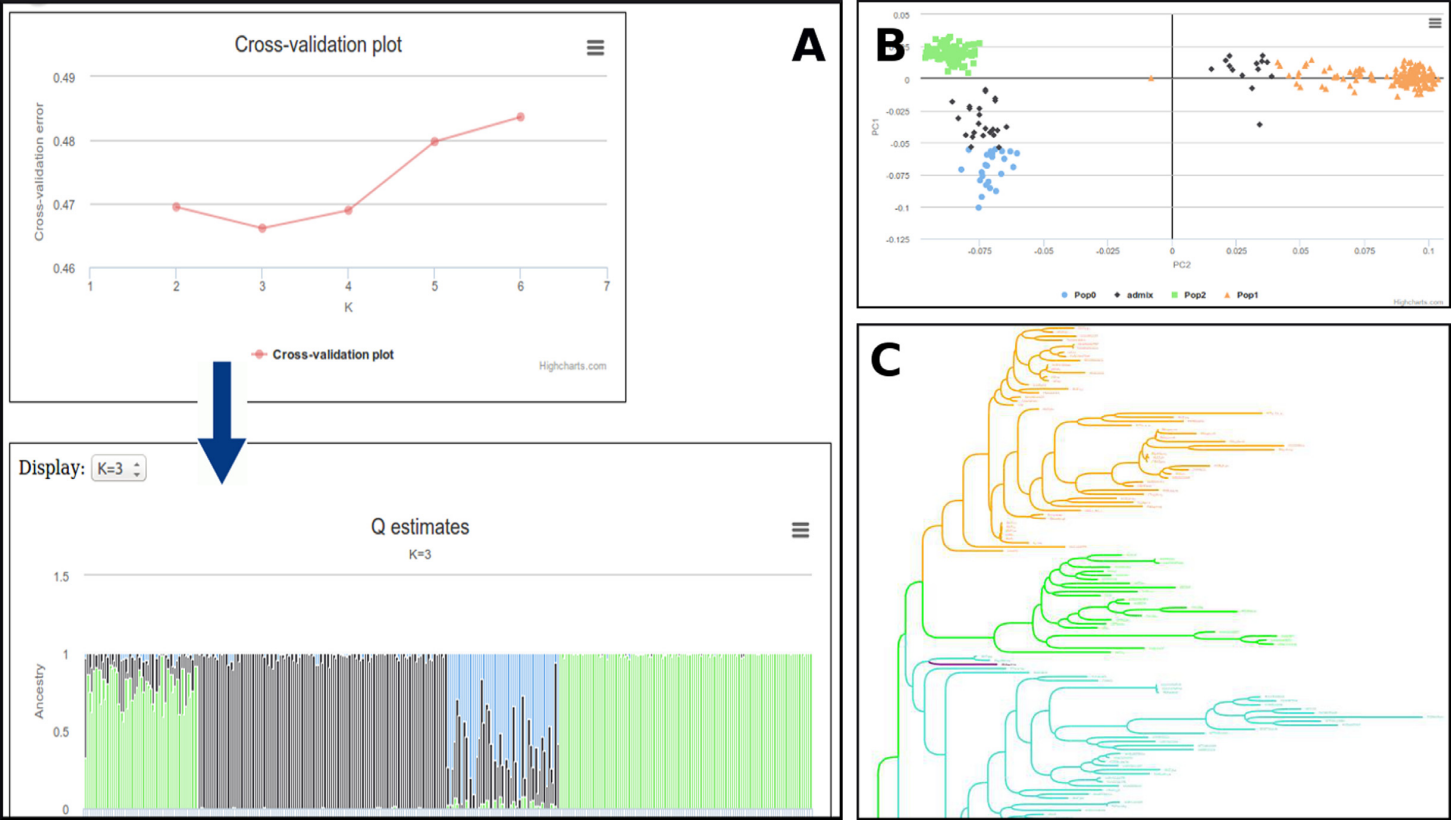
Overview of population structure analyses. (**A**) Structure population inference using the Admixture software. (**B**) Multi-dimensional scaling (MDS) plot representation. (**C**) Colorized and customizable SNP-based distance tree, using InTreeGreat.

### Variant annotation

SnpEff (7) was implemented for variant annotation. When a VCF file is submitted, SnpEff is computed to rapidly categorize the effects of variants in the reference genome sequence selected by the user. SnpEff annotates variants based on their genomic locations (annotated genomic locations can be intronic, exonic or intergenic) and predicts coding effects (mainly synonymous or non-synonymous amino-acid replacement). At the time of writing, about 20 plant genomes (for which genome sequence and Generic File Format (GFF3 annotation file) have been indexed and built into a SnpEff database) are available in the website. The process can be also applied to any other plant, animal or bacteria species, by providing the genome file and its structural annotation. Users can request for a new genome to be added as an available genome database. Following the SnpEff analysis, the process also calculates for each intragenic variant its position within the gene, so that it can be subsequently reintegrated in a FASTA sequence file for diversity analyses on genes for instance.

An option anticipates the situations where the input VCF contains variant positions on the genes instead of chromosomes. This can be the case for RNA-Seq experiments for which reads may have been mapped on coding sequences (CDS or full length mRNAs). This option activates a module that uses the GFF annotation file to locate variants on the chromosomes and recode the VCF file prior to launch SnpEff.

### SNP filtering

By establishing ‘on the fly’ a data matrix combining a subset of individuals and a subset of chromosomes or genes, variants can be filtered out using numerous criteria such as MAF (Minor Allele Frequency), type of variants (SNP or InDel) and missing data ratio. While former version of SNiPlay was built on MySQL database to store and query variants, the version 3 is now based on new technologies or approaches more efficient for the mining of millions of variants at the whole-genome level. For this purpose two methods are thus proposed:

- The first one is based on VCFtools program (6) providing easily accessible methods for working with complex genetic variation data in the form of VCF files. Based on flat files, its binary executable makes it possible to quickly filter out specific variants for a subset of individuals (combining, amongst others, –indv, –chr, –maf parameters).
- The second method takes advantages of a new NoSQL-based application (14) dedicated to big data management. This application, embedded in the SNP filtering interface, optimizes the report of variants in acceptable responding time even for millions of variants. It noticeably enables to discard non relevant or uncertain markers based on depth information, treating as missing data a genotyping point assigned with a read depth under a threshold. Moreover, it restores and recalculates the phasing information after filtering at export time.

Whatever method is chosen, various genotyping file formats can be exported (VCF, plink, ped, hapmap, etc.). In addition, the system also allows the export of FASTA sequences, by re-introducing alleles into flanking sequences extracted from reference genome.

### Population structure analysis

Population stratification represents a major challenge in genome-wide association studies. Admixture (15) is a software tool for maximum likelihood estimation of individual ancestries from multi-locus SNP genotype datasets. It uses the same statistical model as STRUCTURE (16) but calculates estimates much more rapidly using a fast numerical optimization algorithm and is thus more appropriate for integration in a web server. Setting different values of *K* to be tested, *K* being the number of subpopulations in the panel, the server provides the percentage of admixtures (Q matrix) for each *K*-value and plots these Q estimates into comprehensive ready-to-print images. In addition, it also plots the standard error of the cross-validation error estimates so that user can assess the best/correct value for *K* (Figure 2).

As alternatives, other methods can also be used to evaluate population structure such as the multidimensional-scaling (MDS) method. Similarly to the principal-component analysis and in contrast to dendrogram inferring methods that generate hierarchical structures, this technique produces two-dimensional or three- dimensional plots in which the entries are spread according to their relatedness. The MDS analysis in SNiPlay makes use of the plink software (17) and outputs a graphical plot of the studied individuals in two or three dimensions, so that populations can be easily identified.

In addition, to illustrate the panel organization, a distance tree based on SNP data can also be generated from a whole allelic dataset, combining dnadist from the PHYLIP package (18) for distance matrix calculation and FastME (19) for tree reconstruction. Tree visualization is finally allowed by the in-house tree viewer called InTreeGreat.

For both representations (MDS plot or tree), subpopulation attributions derived from the model-based approach are projected and colored on the graphs. An accession is discretely assigned to a subpopulation if more than a user-defined proportion (default: 70%) of its composition came from this subpopulation.

### Genome-wide association studies (GWAS)

**Figure 3.**
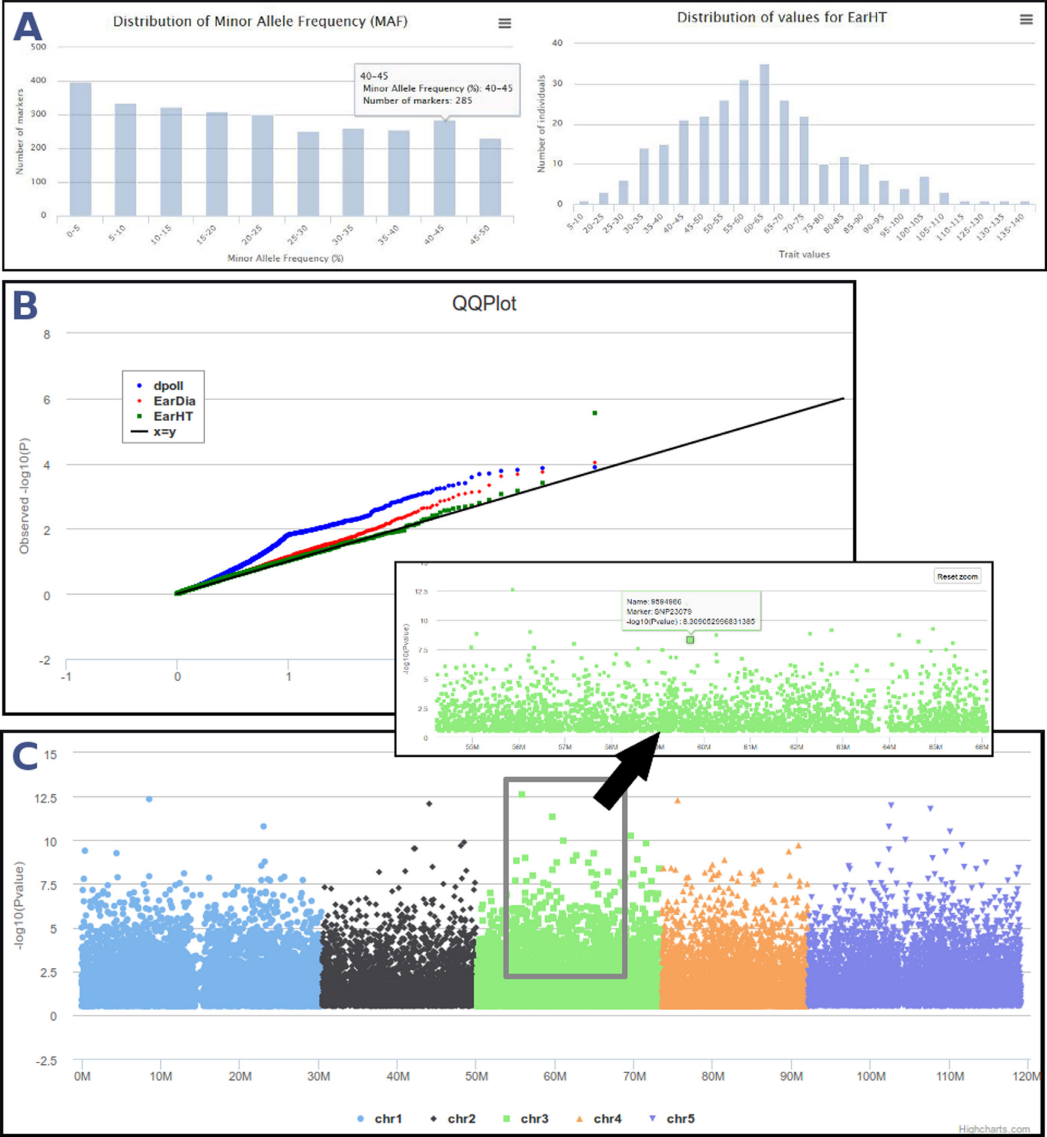
GWAS analyses in SNiPlay. (**A**) Data control: first step controls data concordance and outputs some statistics about genotypic (MAF distribution) and phenotypic (phenotypic values distribution) datasets. (**B**) QQ plot shows the expected distribution of association test statistics (X-axis) compared to the observed values (Y-axis). (**C**) Result interface displays an interactive Manhattan plot color-coded by chromosome that represents the association *P*-values between markers and the trait being measured. It supports zooming, which can be achieved by a ‘click, hold and drag mouse’ action on the region of interest.

GWAS have proven a useful technique for identifying genetic loci involved in the control of agronomic traits. Using an efficient implementation of softwares devoted to GWAS, including TASSEL (20) (General Linear Model (GLM) or Mixed Linear Model (MLM) methods) or MLMM (Multi-Locus Mixed Model) R package (21), phenotypic values of traits can be uploaded and mapped with an accurate model in order to estimate causal variants. This module offers a user-friendly interface that includes interactive Manhattan plots of association *P*-values along the chromosomes, as well as the QQ-plots commonly used for GWAS to show the importance of correcting for population structure and kinship information (Figure 3).

Identifying causal SNPs remains a key challenge in GWAS era, as many variants fall into non-coding regions. For that reason, SNiPlay allows a direct link from any marker presenting a high signal in GWAS results toward genome structural annotations (if available) in JBrowse (22), in order to look at its genomic context. This cross-linking with Genome Browser is possible for every whole genome overview (GWAS, SNP density, diversity in sliding window) if the genome annotation is provided.

### Other facilities

#### General statistics

Using VCFtools, this module provides and displays various statistics about the dataset such as annotation statistics, type of variations or heterosygosity and missing data per sample.

#### SNP density across genome

SNP density can be investigated and compared between different species, subsets of individuals or categories of SNPs (intronic versus exonic for instance). In addition, density of SNPs is also calculated across several samples taken individually and displayed as a heatmap, reflecting thus the difference with the reference genome. This functionality can be of great interest for whole genome structure analysis.

#### Diversity analyses

With the same philosophy, various diversity indices (nucleotide diversity (π), Tajima's D-test of neutral evolution (23), fixation index Fst and Ts/Tv ratio) can be calculated for a predefined size of sliding window using VCFtools and displayed along chromosomes. Moreover, a gene by gene analysis performed by the Egglib libray (24) allows scanning for candidate genes evolving under selection.

#### Haplotype analysis from phased VCF

The VCF format anticipates the coding of allele phasing information (allele pairs specified by 0|1 instead of 0/1 if phased with the previous variant) in order to define haplotype blocks. Thus, if provided in the VCF, a dedicated module of SNiPlay takes advantage of the phasing information to reconstitute and output haplotype blocks of high SNP density regions for which there is a continuum of phasing at the reads level.

## IMPLEMENTATION

SNiPlay is written in Perl; the SNiPlay suite is available as a one-click installation from any local Galaxy instance via the Galaxy Tool Shed (http://toolshed.g2.bx.psu.edu/) or can be downloaded from the SourceForge code source repository (http://sourceforge.net/projects/sniplay/), and installed on a web server. More information on the different installation procedures can be found in the package. The web application is connected to an High Performance Computing (HPC) cluster that manages the jobs sent by the web server (using Opal Perl services and SGE). The install procedure of the application includes a configuration file which has to be adjusted in order to indicate if jobs must be computed locally (by default) or in a remote cluster. Except for tree rendering and haplotype network, all graphical layouts are based on the Highcharts API (http://api.highcharts.com). User data results are conserved for one week on the web server so that users are able to come back and work on their data, by entering the URL including a session identifier. Afterward, user data are removed from the server.

## CONCLUSION

SNiPlay is a flexible and user-friendly web application that rapidly explores and analyzes genetic variants including both SNPs and InDels data. What was possible with the previous release of SNiPlay for a few genes or regions can now be applied for massive genotyping data at the whole-genome level. Indeed, the version 3 allows users to easily manage and exploit SNPs derived from NGS technologies by integrating the standard VCF format as input. In addition to the various large scale analyses allowed by the application like genomic annotation of SNP, diversity analysis, haplotype reconstruction and network, linkage disequilibrium, SNiPlay3 proposes pipelines for GWAS, population stratification, distance tree analysis and visualization of SNP density. Most of the results are displayed using embedded interactive graphical viewers. The complete pipeline can also be deployed on a Galaxy instance using the Galaxy ToolShed.
